# Systemic Effects of a Phage Cocktail on Healthy Weaned Piglets

**DOI:** 10.3390/biology13040271

**Published:** 2024-04-18

**Authors:** Yankun Liu, Yan Lin, Weiyun Zhu

**Affiliations:** 1 Laboratory of Gastrointestinal Microbiology, Jiangsu Key Laboratory of Gastrointestinal Nutrition and Animal Health, College of Animal Science and Technology, Nanjing Agricultural University, Nanjing 210095, China; 2019105078@njau.edu.cn (Y.L.); zhuweiyun@njau.edu.cn (W.Z.); 2 National Center for International Research on Animal Gut Nutrition, Nanjing Agricultural University, Nanjing 210095, China; 3 National Experimental Teaching Demonstration Center of Animal Science, Nanjing Agricultural University, Nanjing 210095, China

**Keywords:** phage cocktail, antioxidant capacity, immune response, intestinal barrier, fecal bacteria community

## Abstract

**Simple Summary:**

Bacteriophages (phages) are viruses specifically infecting bacteria, and have been considered as a prospective substitute for antibiotics. It is necessary to evaluate the impact of phages on overall body health before phage application. This study suggests that the effects of phages on body health are complex, especially regarding immune status. Our results are informative and further improve the implementation and supervision of phage therapy.

**Abstract:**

Numerous studies have demonstrated that bacteriophages (phages) can effectively treat intestinal bacterial infections. However, research on the impact of phages on overall body health once they enter the intestine is limited. This study utilized weaned piglets as subjects to evaluate the systemic effects of an orally administered phage cocktail on their health. Twelve 21-day-old weaned piglets were divided into control (CON) and phage gavage (Phages) groups. The phage cocktail consisted of five lytic phages, targeting *Salmonella enterica* serovar Choleraesuis (*S. choleraesuis*), Enteropathogenic *Escherichia coli* (EPEC), and Shiga tox-in-producing *Escherichia coli* (STEC). The phages group received 10 mL of phage cocktail orally for 20 consecutive days. The results show that the phage gavage did not affect the piglets’ growth performance, serum biochemical indices, or most organ indices, except for the pancreas. However, the impact on the intestine was complex. Firstly, although the pancreatic index decreased, it did not affect the secretion of digestive enzymes in the intestine. Secondly, phages increased the pH of jejunum chyme and relative weight of the ileum, and enhanced intestinal barrier function without affecting the morphology of the intestine. Thirdly, phages did not proliferate in the intestine, but altered the intestinal microbiota structure and increased concentrations of microbial metabolites isobutyric acid and isovaleric acid in the colonic chyme. In addition, phages impacted the immune status, significantly increasing serum IgA, IgG, and IgM, as well as serum and intestinal mucosal IFN-γ, IL-1β, IL-17, and TGF-β, and decreasing IL-4 and IL-10. They also activated toll-like receptors TLR-4 and TLR-9. Apart from an increase in basophil numbers, the counts of other immune cells in the blood did not change. This study indicates that the impact of phages on body health is complex, especially regarding immune status, warranting further attention. Short-term phage gavage did not have significant negative effects on health but could enhance intestinal barrier function.

## 1. Introduction

As one of the greatest discoveries of the last century, antibiotic products have saved countless human and animal lives [1]. However, antibiotic abuse is becoming an increasingly serious problem, leading to drug resistance [2,3]. It is urgent to find supplements or substitutes for antibiotic drugs. Bacteriophages (phages) are viruses that specifically infect bacteria. Depending on the outcome of the infected bacterial host, phages are classified into virulent and temperate types [4]. Virulent phages, in particular, have been utilized as natural antimicrobial agents to control bacterial infections [5]. This approach is known as phage therapy. Unlike antibiotics’ broad-spectrum bactericidal properties, phages target specific hosts, usually one or several strains within a bacterial species [6]. This makes them particularly suitable for the targeted removal of pathogens, especially drug-resistant ones, in places where commensal flora exists, such as the intestine, without disturbing it [7]. Their high specificity can limit their application. However, a phage cocktail, which is a combination of phages, can overcome the limitations of a single phage, such as a narrow host range and the emergence of phage-resistant bacteria [7]. Another advantage of phages is their ability to proliferate by lysing host bacteria, requiring only a small dose to maximize effect (exponential reproduction) [6]. Phage therapy has been applied in human medicine [7,8,9], animal health care [4,10,11], and food safety [12,13,14], achieving convincing results.

Although phages have been used therapeutically in vivo since their discovery, information on the pharmacokinetics of orally administered phages is scarce. As prokaryotic viruses, phages were long thought incapable of infecting eukaryotic cells. However, studies show that phages can enter the internal body environment after mucosal administration [15] and be detected in various tissues and organs, including the brain, via blood transport [16,17,18]. This process may involve various interactions with eukaryotic cells [16,17,18]. These findings increase the uncertainty around phage therapy. As exogenous immunogenic substance, in addition to the expected targeted removal of pathogenic bacteria, the influence of phages on non-target microbial communities and the metazoan host body, especially the immune system, requires further research. This is crucial for the safe in vivo use of phages to control bacterial infections. Therefore, in this study, weaned piglets were used to evaluate the safety of orally administered phage cocktails over 20 consecutive days. We monitored changes in their blood and intestinal indicators, focusing on the immune system and intestinal microbiota. The aim was to deepen our understanding of phages’ interactions with bacterial communities and their metazoan hosts.

## 2. Materials and Methods

### 2.1. Bacteriophages

The phage cocktail in this study consisted of five lytic phages, including four targeting specific pathogenic bacteria and one presumed core phage from pig guts. The specific phages were S19cd (MZ150758, *Felixounavirus*, 85,995 bp), S143_2 (MZ189261, *Mosigvirus*, 168,769 bp), N2 (ON645936, *Tequatrovirus*, 169,736 bp), and C6 (MW679410, *Dhakavirus*, 169,530 bp), targeting *Salmonella enterica* serovar Choleraesuis (*S. choleraesuis*), Enteropathogenic *Escherichia coli* (EPEC), and Shiga toxin-producing *Escherichia coli* (STEC), respectively. Phage C1 (MH717097, *Deseoctovirus*, 46,667 bp), isolated from a pig farm and found in 7/10 pig fecal samples, was considered a core phage in that environment [19], and later shown to be prevalent in Chinese pig guts [20]. The lysis spectrum of the five phages is listed in Table S1.

### 2.2. Animals and Experimental Design

Twelve 21-day-old weaned piglets (6.24 ± 0.46 kg) were randomly assigned to two groups, each with six replicates (1 pig per pen): a control group (CON) and a phages group (Phages). For 20 consecutive days, each piglet received a 10 mL gavage of either the phage cocktail (Phages group) or SM (saline magnesium) buffer (CON group) each morning before feeding. Briefly, a 10 mL phage cocktail was drawn using a 20 mL disposable syringe. After discarding the needle, we attached the syringe to one end of the 25 cm disposable medical enema hose. The other end of the hose was inserted into the esophagus from the pharynx to the stomach and the phage fluid was injected. Five phages were given at an equal ratio (5 × 10^8^ PFU of each phage per 10 mL). Piglets had ad libitum access to feed and water throughout the study, with daily feed consumption recorded. Endotoxin levels in phage preparations and water samples were assessed before the study. The experimental protocol was approved by the Animal Care and Use Committee of Nanjing Agricultural University (SYXK 2020-0066, 16 July 2020), following Chinese animal welfare guidelines. 

### 2.3. Data Recording and Sample Collection

Blood and fresh fecal samples were collected after fasting for 8 h, on the day before the study, and on days 11 and 21 before the morning gavage. Fecal samples were immediately stored at −20 °C. A part of the blood samples was anticoagulated to obtain whole blood and then sent to a hospital for routine blood index analysis the same day. The remainder was left at room temperature for 2 h, then centrifuged at 3000 rpm for 10 min to separate serum. A portion of the serum was analyzed for biochemical indexes at the Animal Hospital of Nanjing Agricultural University the same day; the rest was stored at −80 °C.

Fecal samples were collected 4, 8, 12, and 24 h post-gavage midway through the study, homogenized with SM buffer, and centrifuged (8500 rpm, 5 min). Phage concentrations were then immediately determined using the double-layer plate method [21] to assess excretion dynamics.

On day 21 (after an 8 h fast), piglets were euthanized, and organs such as the heart, liver, spleen, pancreas, and kidney were harvested and weighed. The jejunum, ileum, cecum, and colon were isolated; lengths and weights were recorded. Tissue samples (2 cm) from the front of the jejunum and the middle of the colon were fixed in paraformaldehyde for HE staining and sectioning. 

The pH of chyme from each intestinal segment was measured. The net weight of each intestinal segment was determined after squeezing out the chyme. All the chyme and scraped mucosa samples were stored in liquid nitrogen immediately after sampling. Mucosal and chyme samples from the jejunum and colon were homogenized with SM buffer, and centrifuged at 8500 rpm for 5 min, and phage concentrations were determined by the double-layer method.

### 2.4. Endotoxin Assay

The ToxinSensor™ Chromogenic LAL Endotoxin Assay Kit (GenScript, L00350, Nanjing, China) was utilized to determine endotoxin levels in piglet drinking water, each phage stock solution and serum following the kit’s instructions.

### 2.5. Growth Performance Evaluation

Piglets were weighed the day before the experiment and on the morning of the 21st day after fasting for 8 h. Total body weight gain and average daily gain (ADG) were calculated. Average daily feed intake (ADFI) and feed-to-weight ratio (F/G) were determined from the total feed intake and weight gain during the trial. The organ index (relative weight) was calculated using the formula (%) = (organ weight or length/live weight before slaughter) × 100%.

### 2.6. Serum Antioxidant Capacity 

Commercial kits (Nanjing Jiancheng Bioengineering Institute, Nanjing, China) were used to measure serum antioxidant indices. This included enzyme activities of glutathione peroxidase (GSH-PX, colorimetric method) and catalase (CAT, ammonium molybdate method), serum malondialdehyde (MDA, TBA method) level, and serum total antioxidant capacity (T-AOC, colorimetric method) as per kit instructions.

### 2.7. Intestinal Histomorphology

Intestinal tissue samples were soaked in 4% paraformaldehyde for 24 h, then rinsed with physiological saline to remove chyme residues. After dehydration and paraffin embedding, slices (3 per sample) were prepared. Slices were stained with hematoxylin and eosin and sealed with gum. Three complete crypt-villus units per slice were photographed at 10× or 40× magnification. Villus height and crypt depth were measured using Image-Pro Plus 6.0 software, and the villus-to-crypt ratio was calculated.

### 2.8. Intestinal Chyme Digestive Enzyme Activity Evaluation

Activities of lipase, trypsin, and α-amylase in pig jejunum chyme were assessed using commercial kits (Nanjing Jiancheng Bioengineering Institute, China), and total protein levels were determined using the BCA method according to kit instructions.

### 2.9. The levels of Serum and Intestinal Mucosal Inflammatory Factors, Immunoglobulin and Toll-Like Receptors Determination

Levels of inflammatory factors (IL-1β (interleukin-1β), IL-4, IL-10, IL-17, TNF-α (tumor necrosis factor- α), IFN-γ (Interferon-γ), TGF-β (transforming growth factors β)), Toll-like receptor 4 (TLR-4), TLR-9, and serum immunoglobulins (IgA, IgG, IgM) in serum and intestinal mucosa were measured using ELISA kits (Jiangsu Meimian Industrial Co., Ltd., Yancheng, China). The total protein level was determined by the BCA method following the kit’s guidelines.

### 2.10. Intestinal Barrier Function Evaluation

Levels of tight junction proteins ZO-1 (zonula occludens 1), Claudin-1, and MUC-2 (mucin 2) in jejunum and colon mucosa were measured using the commercial ELISA kit (Jiangsu Meimian Industrial Co., Ltd., Yancheng, China). Two kits (Nanjing Jiancheng Bioengineering Institute, China) were used to assess the levels of D-lactic acid (ELISA method) and DAO (diamine oxidase, colorimetry method) in pig serum, as instructed.

### 2.11. Fecal Bacterial Community Analysis

A commercial Stool DNA kit (Omega BioTek, Norcross, GA, USA) was used to extract fecal DNA, following the provided instructions for specific operations. The extracted DNA was sent to Shanghai Lingen Biotechnology Co., Ltd. (Shanghai, China) for 16S rRNA gene sequencing. Specific primers (341F: 5′-CCTAYGGGRBGCASCAG-3′; 806R: 5′-GGACTACNNGGGTATCTAAT-3′) were used to amplify the V3-V4 region of 16S rDNA. Sequencing results, including the operational taxonomic units (OTUs) data, were uploaded to www.microbiomeanalyst.ca (accessed on 5 December 2023) for analysis. This process facilitated the analysis of microbial community composition at each taxonomic level, α and β diversity, and the differential bacteria between groups. The raw reads of 16s rDNA of fecal microbiota were deposited into the NCBI Sequence Read Archive (SRA) database (accession number: PRJNA1088713).

### 2.12. Intestinal Chyme Short-Chain Fatty Acids (SCFAs) Determination

A 0.4 g sample of colon or cecum chyme was mixed with 1.6 mL double distilled water, vortexed for homogenization, and then centrifuged at 13,400× *g* for 10 min. The supernatant was combined with a mixture solution (0.6464 g crotonic acid dissolved in 25% (*w*/*v*) metaphosphoric acid) in a new centrifuge tube at a 5:1 ratio, mixed evenly, and placed in a −20 °C freezer. After 12 h, the mixture was thawed and centrifuged at 13,400× *g* for 10 min at room temperature. The supernatant was filtered through a 0.22 μm filter membrane. Filtrate and ether were mixed in a new centrifuge tube at a 1:1 ratio (Extraction). The upper liquid was then used to measure SCFAs with a gas chromatograph (Thermo Scientific, Waltham, MA, USA, trace 1310).

### 2.13. Statistical Analysis

Data, except for those regarding the relative abundance of bacteria, are presented as “mean ± standard error of the mean (SEM)”. The independent sample *t*-test in SPSS 26 was used to analyze differences between groups. The relative abundance of bacteria is shown as “median ± quartile”. The Mann–Whitney U test in SPSS 26 helped identify differential bacteria between groups. Data with a *p*-value < 0.05 were considered significant. All figures were created with GraphPad Prism 8.

## 3. Results

### 3.1. Endotoxin Levels in Phage Stock Solutions and Drinking Water

As indicated in Table 1, the total endotoxin level in the phage cocktail administered orally was 26,890.21 ± 846.20 EU daily. Weaned piglets consume approximately 1.5–2.5 L of water per day using nipple-type automatic drinking fountains [22]. Assuming a daily water consumption of 2 L for a piglet, the endotoxin intake through water in the control group was 144,920.00 ± 9364.81 EU. In contrast, the phages group’s daily endotoxin intake from drinking water and phage gavage was 171,810.21 ± 8950.55 EU. There was no significant difference in daily endotoxin intake between the two groups (*p* > 0.05).

### 3.2. Fecal and Intestinal Phage Quantification

**Figure 1:**
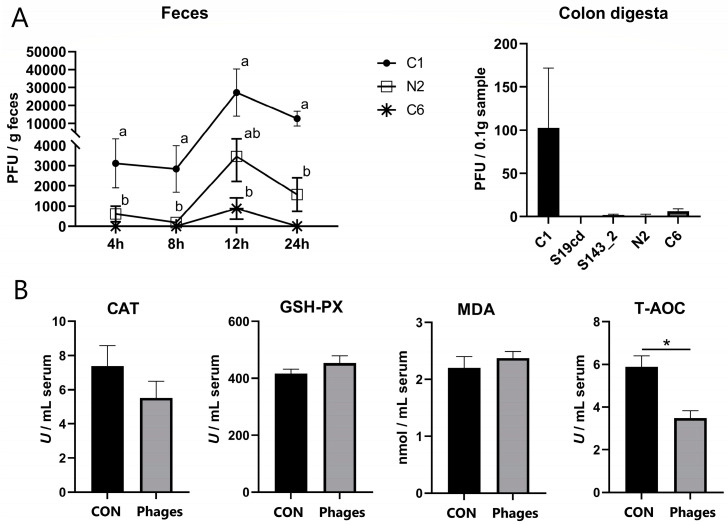
Quantification of phages in feces and colon digesta (**A**) and effects of phage cocktail on the serum antioxidant capacity of piglets (**B**). Note: Different letters mean a significant difference between the groups (*p* < 0.05), while no letter or the same letters mean no significant difference; * represents a significant difference (*p* < 0.05).

The peak of phage excretion for each strain occurred at 12 h after gavage, as shown in Figure 1A. Phages S19cd and S143_2 were scarcely detected at any time point, and phage C6 was found at 4, 8, and 24 h, but only in the feces of a few pigs. The fecal excretion of phage C1 exceeded that of N2 at 4, 8, and 24 h (*p* < 0.05) and surpassed C6 at 12 h (*p* < 0.05). Within 24 h, the excretion of all phage strains was significantly reduced from the initial gavage (5 × 10^8^ PFU/strain), indicating a lack of host bacteria in the intestine and no proliferation of the phages. Phage quantification in intestinal chyme after slaughter revealed a complete absence of all five phages in jejunal chyme. Only a trace of phage C1 was found in colonic chyme, with other phages almost absent or detected in only a few pigs (Figure 1A).

### 3.3. Effects of Phage Cocktail on Growth Performance, Routine Blood Parameters, and Antioxidant Capacity of Piglets

Weaned piglets, due to their underdeveloped digestive and immune systems, are vulnerable to environmental factors and experimental treatments, often reflected in their suboptimal growth performance. The administration of a phage cocktail intragastrically for 20 days did not notably influence the growth performance of piglets, including ADG (average daily gain), ADFI (average daily feed intake), and F/G (feed-to-weight ratio) (Table S2). 

The continuous administration of the phage cocktail for 10 days did not alter serum biochemical and blood routine indicators of piglets (Tables S3 and S4). However, after 20 days, the concentration and proportion of basophilic granulocytes (Bas) were significantly higher in the phages group (*p* < 0.05), with no significant differences in other indicators between the two groups (Tables S3 and S4). According to serum antioxidant indicators (Figure 1B), T-AOC (total antioxidant capacity) in the phages group was significantly lower than in the control group after 20 days of gavage (*p* < 0.05). Other antioxidant indicators, including GSH-PX (glutathione peroxidase) and CAT (catalase) activity and MDA (malondialdehyde) concentration, showed no significant difference between the two groups (*p* > 0.05). 

### 3.4. Effects of Phage Cocktail on Intestinal Digesta pH, Digestive Enzyme Activity, and Intestinal Morphology of Piglets

The organ index after slaughter on day 21 showed that the relative weight of the pancreas in the phages group was significantly lower than in the control group (*p* < 0.05). The relative net weight of the ileum was significantly increased (*p* < 0.05). No significant differences were observed in other organ indices between the two groups (*p* > 0.05) (Tables S5 and S6). 

The intestinal chyme pH of piglets was measured after slaughter at 21 days. The results indicate (Table 2) that, compared with the control group, the jejunum chyme pH in the phages group was significantly higher (*p* < 0.05). This difference was not observed in the cecum and colon chyme (*p* > 0.05). Given the significantly decreased pancreatic index in the phages group, the activities of digestive enzymes secreted by the pancreas were analyzed. There were no significant differences in the activities of lipase, trypsin, and amylase between the two groups (*p* > 0.05) (Table S7). Additionally, intestinal histomorphology results showed no significant differences in jejunum villi height, crypt depth, or villi-crypt ratio between the two groups (*p* > 0.05) (Table S8). These findings indicate that although jejunum pH increased significantly, digestive enzyme activity and intestinal morphology remained unchanged. Thus, the phage cocktail had no significant effect on the digestive-related indices of piglets.

### 3.5. Effects of Phage Cocktail on Inflammatory Cytokines and Immunoglobulin Levels in Piglets

**Figure 2:**
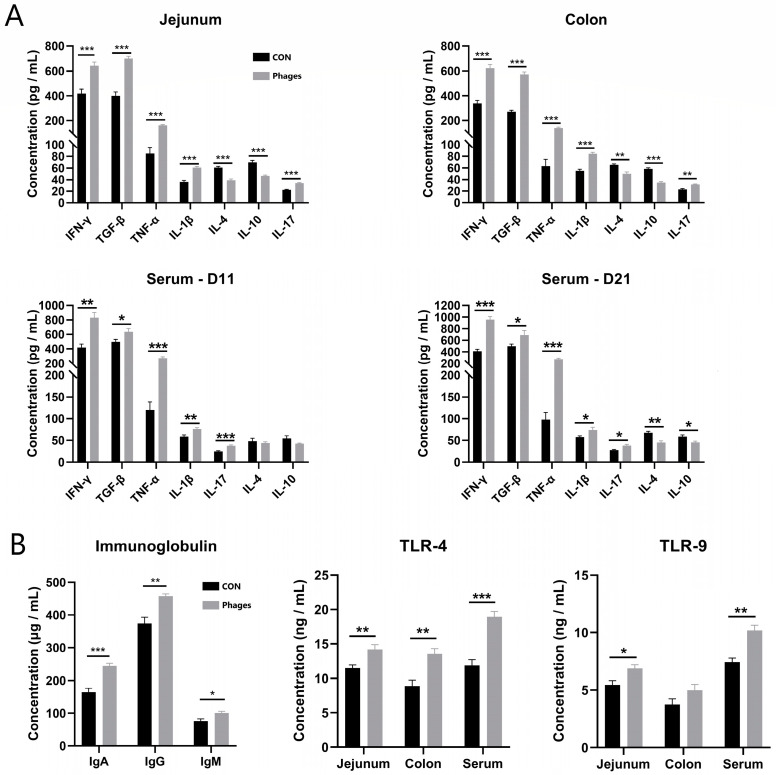
Effects of phage cocktail on the levels of inflammatory cytokine (**A**), Ig and TLR receptors (**B**) in serum and intestinal mucosa of piglets. Note: * represents *p* < 0.05, ** represents *p* < 0.01, and *** represents *p* < 0.001. The same is applied to other figures.

The intestinal mucosa was scraped to quantify inflammatory factors. The data in Figure 2A reveal that the levels of pro-inflammatory factors in the jejunal and colonic mucosa of the phages group, including IL-1β (interleukin-1β), IL-17, TNF-α (tumor necrosis factor-α), and IFN-γ (Interferon-γ), were significantly increased (*p* < 0.01). Conversely, the levels of the anti-inflammatory factors IL-10 and IL-4 were significantly lower than in the control group (*p* < 0.01). However, the levels of another anti-inflammatory factor, TGF-β (transforming growth factors β), were significantly higher than in the control group (*p* < 0.01). The serum inflammatory level was consistent with the findings in the intestinal mucosa.

Furthermore, the levels of serum IgA (immunoglobulins A), IgG, and IgM in the phages group were also significantly higher than in the control group after 20 days of gavage (Figure 2B; *p* < 0.05). These results suggest that the phage cocktail elicited a humoral immune response in piglets. Hence, the level of pattern recognition receptors, which specifically recognize pathogens in the serum and intestinal mucosa, was analyzed. The ELISA quantitative results (Figure 2B) show that after 20 days of continuous gavage, the TLR-4 (Toll-like receptor 4) and TLR-9 levels in the jejunum mucosa and serum and TLR-4 in the colon mucosa of the phages group were significantly higher than those in the control group (*p* < 0.05). This indicates that the phage and its nucleic acid may be recognized by TLR receptors of immune cells, triggering immune reactions such as the production of downstream inflammatory factors.

### 3.6. Effects of Phage Cocktails on Intestinal Barrier-Related Indices in Piglets

**Figure 3:**
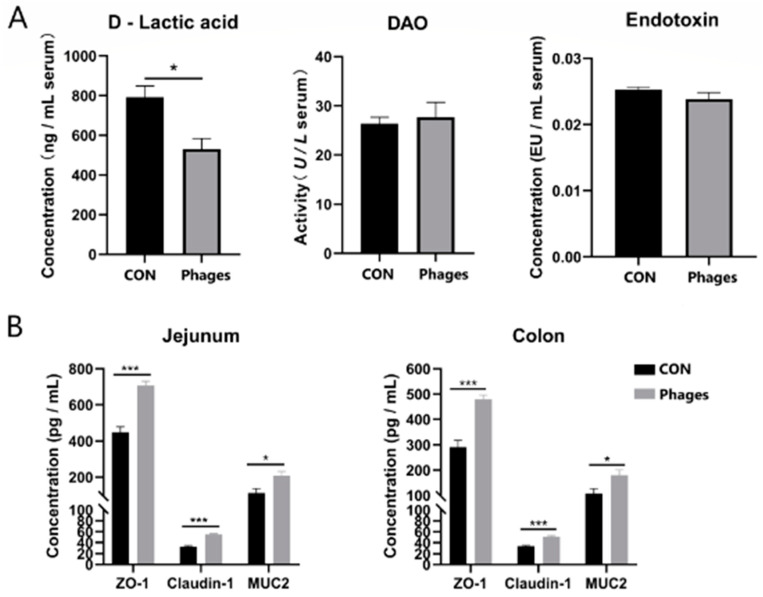
Effects of phage cocktail on the levels of DAO, D-lactic acid, endotoxin in serum (**A**) and tight junction proteins in intestinal mucosa (**B**) of piglets. Note: * represents *p* < 0.05 and *** represents *p* < 0.001. The same is applied to other figures.

As shown in Figure 3A, after 20 consecutive days of gavage, no significant differences were observed in serum DAO (diamine oxidase) activity or serum endotoxin levels between the two groups (*p* > 0.05). However, the serum D-lactic acid level in the phages group was significantly lower than in the control group (*p* < 0.05). Coinciding with this, the ELISA quantification of tight junction proteins in the jejunum and colon mucosa (Figure 3B) revealed that the levels of ZO-1 (zonula occludens 1), Claudin-1, and mucin 2 (MUC2) in the phages group were significantly higher than in the control group (*p* < 0.05). Combining these results suggests that the intragastric administration of phages may enhance the intestinal barrier function of piglets.

### 3.7. Effects of Phage Cocktail on Fecal Bacteria Community 

**Figure 4:**
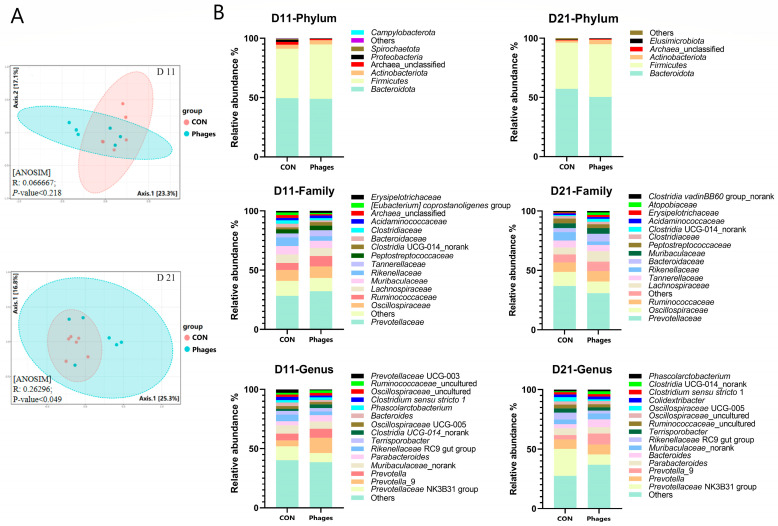
Effects of phage cocktail on fecal bacteria community. (**A**) Principal coordinate analysis (PCoA) of fecal microbiota based on Bray-Curtis distance. (**B**) The composition of the gut microbiota at various taxonomic levels. Note: Only the top 15 bacteria families and genera are displayed at the family and genus levels.

After continuous gavage, there were no significant differences in the α-diversity of fecal microbiota between the two groups (*p* > 0.05) (Table S9). However, the PCoA analysis (principal coordinate analysis) base on the Bray–Curtis distance algorithm revealed (Figure 4A) that the phages group significantly diverged from the control group on the 21st day (*p* < 0.05). This divergence indicates that the microbial structure of the piglets underwent significant changes after phage gavage, despite showing no differences at day 11 (*p* > 0.05).

**Figure 5:**
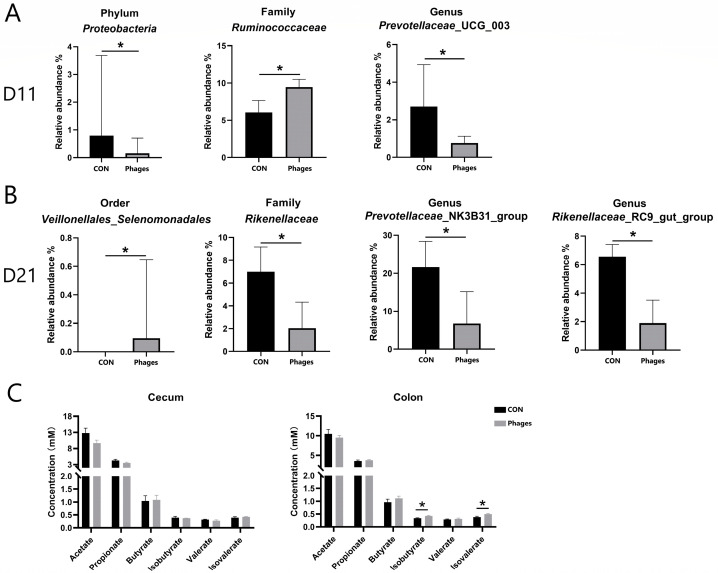
Analysis of differential bacteria (**A**,**B**) and bacterial metabolites (**C**). Note: * represents *p* < 0.05.

As shown in Figure 4B, at day 11 and day 21, *Bacteroidota* and *Firmicutes* remained the dominant phyla in both groups, followed by *Actinobacteriota*. The top three families were *Prevotellaceae*, *Oscillospiraceae*, and *Ruminococcaceae*. On the 11th day, at the phylum level (Figure 5A), the relative abundance of *Proteobacteria* in the phages group was significantly lower than in the control group (*p* < 0.05). At the family (top 20) and genus (top 30) levels, the relative abundance of *Ruminococcaceae* increased significantly, and *Prevotellaceae*_UCG_003 decreased significantly in the phages group (*p* < 0.05). On the 21st day (Figure 5B), at the order level, the relative abundance of *Veillonellales*_*Selenomonadales* in the phages group saw a significant increase (*p* < 0.05). At the family level (top 20), the relative abundance of *Rikenellaceae* was significantly reduced in the phages group (*p* < 0.05), with this difference extending to the significant decrease in *Rikenellaceae*_RC9_gut_group at the genus level (*p* < 0.05). Additionally, at the genus level, the relative abundance of the *Prevotellaceae* _NK3B31_group in the phages group also saw a significant decrease (*p* < 0.05).

Regarding the quantitative analysis of bacterial metabolite short-chain fatty acids (SCFAs) in large intestine chyme (Figure 5C), there were no significant differences in the levels of various SCFAs in cecal chyme between the two groups (*p* > 0.05). However, the concentrations of isobutyric and isovaleric acids in colon chyme were significantly higher in the phages group compared to the control group (*p* < 0.05).

## 4. Discussion

Phage is an important member of the microbial community in human and animal intestines. Although phages are believed to be incapable of infecting eukaryotic cells, recent research on phage therapy has shown that phages can be detected in the blood through mucosal delivery methods (such as oral and intranasal administration). From there, they can transfer to tissues and organs throughout the body via the blood, such as the spleen, liver, kidney, and even the brain [16,17,18,23]. Therefore, the impact of phage preparations on the body is no longer limited to the direct elimination of pathogenic bacteria. The potential effects on routine health indicators and immune status are also significant.

### 4.1. Most Phages Are Deactivated in the Gastrointestinal Passage 

In this study, over time, the number of phages excreted and retained continuously decreased. This indicates a lack of corresponding host bacteria in the piglet intestinal tract, preventing these phage strains from proliferating. Furthermore, we initially administered phages in an amount of 5 × 10^8^ PFU. However, in the feces, we detected only about 10^7^ PFU (daily defecation of a post-weaned piglet is about 50–100 g), suggesting more than 98% of administered phages were deactivated after passing through the gastrointestinal tract. This is due to factors such as the low pH value of the stomach, pancreatic digestive enzymes, and bile salts in the small intestine, which may reduce the viability/stability of phages [24]. Phage resilience varies, such as C1 having better endurance than the other four phages. Thus, it is considered that phage therapy for prevention requires repeated doses to ensure sustained presence in the gut [25] or encapsulation to deliver most phages to the posterior digestive tract [26].

### 4.2. Phages Can Alter the Host Immune Status of Piglets

Our results show that administering a cocktail of phages by gavage stimulates a low-grade homeostatic immune response. This is evident as it did not affect the numbers of white blood cells, neutrophils, eosinophils, monocytes, or lymphocytes in the blood. It only caused a slight increase in basophils and less than a twofold increase in pro-inflammatory factor levels. Additionally, while some pro-inflammatory factors increased, the anti-inflammatory factor TGF-β also improved. TGF-β is a cytokine with immunosuppressive activity produced by various cells. It maintains immune homeostasis by promoting the differentiation of regulatory T cells (Treg). This regulates or inhibits the production of pro-inflammatory cytokine IL-17 by helper T cell 17 (Th17) [27,28]. In this study, piglets may have moderated the production of pro-inflammatory factors by increasing TGF-β secretion, thus regulating the intestinal mucosa and overall immunity. Numerous studies have reported phages triggering low-level immune responses [29,30,31,32]. Phages entering the intestine might cross the intestinal mucosa and enter the lamina propria, stimulating mucosa-associated lymphoid tissue and impacting immune cells in other parts of the body via the bloodstream [15]. In response, the body may eliminate the phages through specific antibodies [33]. Majewska et al. discovered that the long-term addition of high-titer T4 phage to drinking water induced intestinal secretory IgA and serum IgG secretion in mice, indicating T4 transfer from the intestinal cavity to the blood circulation system [16]. This study also found an increase in serum Ig levels after phage administration, suggesting an activation of humoral immunity. Furthermore, epithelial and immune cells can recognize phage proteins and nucleic acids through the endocytic pathway [33]. Degraded nucleic acids are detected by TLRs [34] (such as TLR-9 [29] and TLR-3 [35], etc.). A significant increase in TLR4 and TLR9 in the intestinal mucosa and serum after phage administration suggests that inflammatory factor production could be triggered by phages entering the circulatory system and being processed by immune cells.

### 4.3. Phages May Promote the Intestinal Health of Piglets

Our results show that the intestinal barrier function of piglets was enhanced after the oral administration of phages, consistently with previous studies [30,36,37]. Cadwell et al. reported that cells internalize phages and use them as a resource to enhance cellular growth and survival [38]. Studies also revealed that TGF-β plays a protective role in intestinal barrier function [39], and IL-17 can induce the expression of tight junction proteins claudin 1 and claudin 2 [40]. We speculate that the enhanced barrier function results from cell internalization of phages and significantly elevated levels of TGF-β and IL-17 in the mucosa. Furthermore, Brabec et al. demonstrated that IL-17 protects epithelial barriers by inducing antimicrobial peptide secretion, protecting against microbiota dysbiosis and inflammation in the ileum [41]. We will further explore whether antimicrobial peptide levels increase in the intestines of piglets after phage administration.

### 4.4. Phages Alter the Intestinal Microbiota Structure Even without Host Bacteria 

Our results indicate that phages did not proliferate in the intestine, meaning they did not encounter their host bacteria, yet they could affect the structure of the intestinal microbiota. How do phages alter the intestinal microbiota without encountering their host bacteria? We propose three possible explanations. First, phages alter the body’s immune status, which inevitably affects bacteria–body interactions and the microbiota structure [42]. Second, phages may increase intestinal antimicrobial peptide secretion [41], influencing some bacteria’s survival and the microbiota structure. Third, a “lysis from without” effect exists, where, although they cannot proliferate in the bacteria, phages bind to bacterial surfaces and punch holes in their cell walls, causing bacterial death [43]. 

A change in the structure of the intestinal microbial community will also lead to changes in bacterial metabolic products. Our study also found a change in SCFA concentration in the intestine. Compared to the control group, the acetic acid content in the cecal chyme of the phages group decreased by 24% (*p* = 0.09). This decrease is likely due to significant reductions in the relative abundances of members of the *Prevotellaceae* and *Rikenellaceae* [44,45]. Intestinal branched-chain fatty acids, such as isobutyric and isovaleric acids, are mainly produced by the microbial fermentation of proteins [46]. The significant increase in isobutyric and isovaleric acid levels in the colonic chyme of piglets in the phages group might result from increased protein-fermenting microbes due to phage administration.

### 4.5. The Complex Health Effects of Phages Require More Research

Our research found that feeding phages to piglets had no effect on the growth performance, but negatively impacted their antioxidant system. Some articles have reported that the phagocytosis of phages into the internal environment often leads to an increase in reactive oxygen species [47,48]. This could explain the reduced antioxidant capacity observed in our study. Additionally, feeding phages to piglets decreased their pancreatic index but did not influence the pancreatic secretion of digestive enzymes. Phages caused an increase in some pro-inflammatory factors in the intestine, but also strengthened this organ’s barrier function. Feeding phages to piglets increased the relative net weight of the ileum and raised the pH level of the jejunum chyme. All these results indicate that phages exert complex effects on piglets. Currently, it is difficult to assess whether feeding phages to metazoan hosts is beneficial or harmful, and this requires more study.

## 5. Conclusions

In the future, bacteriophage therapy may emerge as an alternative to antibiotic treatments, underscoring the critical importance of safety assessments for bacteriophage use. This study discovered that a short-term, 20-day regimen of oral phage administration does not affect the growth performance of piglets or cause significant negative effects on their health. However, the implications for intestinal health and immune status are complex and demand further examination.

## Figures and Tables

**Table 1:** Endotoxin level in phage stock solutions and drinking water.

Phage Strain	Endotoxin Concentration (EU/mL)	Average Daily Gavage Volume (μL)
C1	96,794.73 ± 4038.27	12
S19cd	39,451.71 ± 1149.41	148.53
S143_2	72,135.35 ± 2369.81	85.88
C6	11,043.06 ± 584.79	147.53
N2	61,158.41 ± 1109.76	196.94
water	72.46 ± 3.82	\

**Table 2:** Effect of phage cocktail on the pH of intestinal digesta.

Segment	CON Group	Phages Group	*p*-Value
Jejunum	6.52 + 0.15 ^a^	6.93 + 0.08 ^b^	0.030
Cecum	6.57 + 0.19	6.64 + 0.09	0.751
Colon	6.50 + 0.16	6.61 + 0.08	0.538

Note: Different superscript letters in the same row indicate significant differences between groups (*p* < 0.05).

## Data Availability

The genome sequences of the five phages (S19cd, S143_2, N2, C6 and C1) were deposited at GenBank (MZ150758, MZ189261, ON645936, MW679410 and MH717097). The raw reads of 16s rDNA of fecal microbiota were deposited into the NCBI Sequence Read Archive (SRA) database (accession number: PRJNA1088713). The raw data supporting the conclusions of this article will be made available by the corresponding author on reasonable request.

## Supplementary Materials

The following supporting information can be downloaded at: https://www.mdpi.com/article/10.3390/biology13040271/s1. Table S1. The lysis spectrum of five phages in the cocktail; Table S2. Effects of phage cocktail on the growth performance of piglets; Table S3. Effects of phage cocktail on the serum biochemical indices of piglets; Table S4. Effects of phage cocktail on the blood routine indices of piglets; Table S5. Effects of phage cocktail on the relative weight of organs in piglets; Table S6. Effects of phage cocktail on the relative weight and length of different intestinal segments of piglets; Table S7. Effects of phage cocktail on the digestive enzymes’ activity of jejunal digesta; Table S8. Effects of phage cocktail on the intestinal morphology of piglets; Table S9. Effects of phage cocktail on the α diversity of fecal microbiota of piglets.
