# Supplementary material for: Systemic Effects of a Phage Cocktail on Healthy Weaned Piglets
Source: Biology (Basel). 2024 Apr 18;13(4):271. doi: 10.3390/biology13040271 (PMC11048100; doi:10.3390/biology13040271)
Supplement: Supplementary file 1 [file biology-13-00271-s001.zip › biology-2944261-supplementary.pdf]

# Supplemental material

Table S1. The lysis spectrum of five phages in the cocktail

| Strains | Accession No. | Antibiotic Resistance*[1] | C1 | S19cd*[2] | 143_2 | N2 | C6 |
|---|---|---|---|---|---|---|---|
| *E.coli* 11 | MH671408 | AMP, TE | | | | | |
| *E.coli* 12 | MH671409 | AMP, TE, C, CN | | | √ | | |
| *E.coli* 13 | MH671410 | AMP, TE, C, CN, ENR, EFT | | | √ | | √ |
| *E.coli* 14 | MH671411 | AMP, TE, C, CN, ENR, EFT | | | | | |
| *E.coli* 15 | MH671412 | AMP, TE, C | | | | | |
| *E.coli* 22 | MH671413 | AMP, TE, C, CN | | | √ | √ | √ |
| *E.coli* 24 | MH671414 | AMP, TE, C, CN, ENR | | | | | |
| *E.coli* 31 | MH671415 | AMP, TE, C | | | | | |
| *E.coli* 33 | MH671416 | AMP, TE, ENR | √ | | | | |
| *E.coli* 34 | MH671417 | AMP, TE, C | | | | | |
| *E.coli* 36 | MH671419 | AMP, TE, C, ENR, EFT | | | | | |
| *E.coli* 37 | MH671420 | None | | | | | |
| *E.coli* 38 | MH671421 | AMP, TE, EFT | | | √ | √ | |
| *E.coli* 41 | MH671422 | AMP, TE, CN, ENR | | | | | |
| *E.coli* 42 | MH671423 | AMP, TE, C, CN | √ | | | | |
| *E.coli* 44 | MH671425 | AMP, TE, C, CN | | √ | | | √ |
| *E.coli* 45 | MH671426 | AMP, TE, C, CN, ENR | | | | | √ |
| *E.coli* 46 | MH671427 | AMP, TE, C | | | √ | | |
| *E.coli* 47 | MH671428 | AMP, TE, CN, EFT | | | | | |
| *E.coli* 51 | MH671429 | AMP, TE, C | | | | | √ |
| *E.coli* 52 | MH671430 | AMP, TE, C, CN, ENR | | | | | |
| *E.coli* 54 | MH671431 | AMP, TE, C, CN | | | | | |
| *E.coli* 56 | MH671432 | AMP, TE, C | | | | | |
| *E.coli* 57 | MH671433 | AMP, TE, C, CN, ENR | | | √ | | |
| *E.coli* 58 | MH671434 | AMP, TE, C, CN | | | | √ | |
| *E.coli* 61 | MH671435 | AMP, TE, C, ENR | | | | | |
| *E.coli* 62 | MH671436 | AMP, TE, CN, ENR | | | √ | | |
| *E.coli* 63 | MK156384 | AMP, TE, C, CN | | | | | |
| *E.coli* 71 | MH671437 | AMP, TE, C, CN, ENR | | | | | |
| *E.coli* 72 | MH671438 | AMP, TE, C, CN, ENR, EFT | | | √ | | |
| *E.coli* 73 | MH671439 | AMP, TE, C, CN, ENR | | | | | |
| *E.coli* 81 | MH671440 | AMP, TE, CN | | | √ | | |
| *E.coli* 82 | MH671441 | AMP, TE, CN, ENR | | | | | |
| *E.coli* 83 | MH671442 | AMP, TE, C, CN, ENR | | | | | |
| *E.coli* 84 | MH671443 | AMP, TE, CN | | | | | |
| *E.coli* 85 | MH671444 | AMP, TE, CN, ENR | | | | | |
| *E.coli* 86 | MH671445 | AMP, TE, C | | | | √ | √ |
| *E.coli* 87 | MH671446 | AMP, TE, CN, ENR | | | | | |
| *E.coli* 91 | MH671447 | AMP, TE, CN | | | √ | | |

| | | | | | | | |
|---|---|---|---|---|---|---|---|
| *E.coli* 94 | MH671448 | AMP, TE, C, CN | | | | | |
| *E.coli* 95 | MH671449 | AMP, TE, CN | | | | | |
| ETEC 101 | MH671450 | AMP, TE, C | | | | | |
| ETEC 102 | MH671451 | TE | | | | | |
| ETEC 103 | MH671452 | AMP, TE, C, CN, ENR | | | | | |
| ETEC 104 | MH671453 | AMP, TE | | | √ | √ | √ |
| ETEC 105 | MH671454 | TE, C, CN, ENR | | | | | |
| ETEC 106 | MK615932 | None | | | | √ | √ |
| *E.coli* 121 | MH671455 | AMP, TE, CN, ENR | | | √ | | |
| *E.coli* 122 | MH671456 | AMP, TE, C, CN, EFT | √ | | | | |
| *E.coli* 123 | MH671457 | AMP, TE, C | | | | | |
| *E.coli* 131 | MH671458 | AMP, TE, C, CN, EFT | √ | | | | |
| *E.coli* 132 | MH671459 | AMP | | | | | |
| *E.coli* 133 | MH671460 | AMP, TE, C, CN, ENR | | | | | |
| *E.coli* 141 | MH671461 | AMP, C | | | | | |
| *E.coli* 142 | MH671462 | AMP, TE, C, CN, ENR | | | | | |
| *E.coli* 143 | MH671463 | AMP, TE, C, CN | | | √ | | √ |
| *E.coli* 145 | MH671465 | AMP, TE, C, CN, EFT | √ | | | | |
| *E.coli* 151 | MH671466 | AMP, TE, C, ENR, EFT | | | | | |
| *E.coli* 156 | MH671467 | AMP | | | | | |
| *E.coli* 161 | MH671468 | CN | | | | | |
| *E.coli* 162 | MH671469 | AMP, TE, C, CN, ENR | | | √ | √ | |
| *E.coli* 164 | MH671470 | AMP, TE, C, CN, EFT | √ | | | | |
| *E.coli* 171 | MH671471 | AMP, TE, C, CN, ENR | | | | | |
| *E.coli* 172 | MH671472 | AMP, TE, C | | √ | √ | | √ |
| *E.coli* 173 | MH671473 | AMP, TE, C, EFT | | | | | |
| *E.coli* 174 | MH671474 | AMP, TE, CN | | | | | |
| *E.coli* 181 | MH671475 | AMP, TE, C, CN, ENR | | | √ | | |
| *E.coli* 182 | MH671476 | AMP, TE, C | | | | | |
| *E.coli* 191 | MH671477 | AMP, TE, C, CN | | | √ | √ | |
| *E.coli* 192 | MH671478 | AMP, TE, C, CN, EFT | √ | | √ | | |
| *E.coli* 193 | MH671479 | AMP, TE, C | | | √ | | |
| *E.coli* 202 | MH671481 | AMP, TE, C, ENR | | | | | |
| *E.coli* 203 | MH671482 | AMP, TE, C, ENR, EFT | | | | | |
| *E.coli* 211 | MH671483 | AMP, TE | | | | | |
| *E.coli* 212 | MH671484 | AMP, TE, CN, ENR | | | | | |
| *E.coli* 231 | MH671486 | TE, C, CN, ENR, EFT | | | | | |
| *E.coli* 232 | MH671487 | AMP, TE, C, CN, ENR | | | | | |
| *E.coli* 233 | MH671488 | AMP, TE, C, CN, ENR, EFT | | | | | |
| *E.coli* 234 | MH671489 | AMP, TE, C | | | | | |
| *E.coli* 235 | MH671490 | AMP, TE, C | | | | √ | |
| *E.coli* 236 | MH671491 | AMP, TE, C, ENR, EFT | | | √ | | |
| *E.coli* 237 | MH671492 | AMP, TE, C, ENR, EFT | | | √ | √ | √ |

| | | | | | |
|---|---|---|---|---|---|
| *E.coli* 241 | MH671493 | AMP, TE, C, CN, ENR | | | √ |
| *E.coli* 242 | MH671494 | AMP, TE, C, ENR, EFT | √ | √ | √ |
| *E.coli* 243 | MH671495 | AMP, TE, C | | | |
| *E.coli* 244 | MH671496 | AMP, TE | | | |
| *E.coli* 245 | MH671497 | AMP, TE, C, CN, ENR | | | |
| *E.coli* W1 | MN086362 | AMP, TE, C, CN, ENR, EFT | | | |
| *E.coli* W3 | MN086363 | AMP, TE, C, CN, ENR, EFT | | | |
| *E.coli* W5 | MN086364 | AMP, TE, C, CN, ENR, EFT | √ | | |

Note: *1, The antibiotics used for resistance tests were gentamicin (CN), ampicillin (AMP), ceftiofur (EFT), tetracycline (TE), chloramphenicol (C) and enoxacin (ENR); *2, S19cd can also infect two pathogenic bacterial strains (ATCC 13312 and CICC 21493) of *Salmonella enterica* serovar Choleraesuis.

Table S2. Effects of phage cocktail on the growth performance of piglets

| Indexes | CON | Phage | *P*-value |
|---|---|---|---|
| Initial body weight (kg) | 6.26±0.11 | 6.56±0.18 | 0.190 |
| Terminal body weight (kg) | 14.23±0.36 | 15.05±0.50 | 0.212 |
| ADG (g/d) | 394.25±14.41 | 424.58±20.19 | 0.249 |
| ADFI (g/d) | 640.30±70.02 | 731.19±55.31 | 0.332 |
| Feed intake/Gain | 1.71±0.07 | 1.76±0.08 | 0.687 |

Table S3. Effects of phage cocktail on the serum biochemical indices of piglets

| Indexes | Day 11 | | | Day 21 | | |
|---|---|---|---|---|---|---|
| | CON | Phage | *P*-value | CON | Phage | *P*-value |
| GLU (mmol/L) | 5.68±0.31 | 4.31±0.74 | 0.131 | 5.76±0.58 | 6.60±0.57 | 0.327 |
| ALB (g/L) | 24.08±0.51 | 22.92±0.69 | 0.205 | 21.25±1.84 | 22.43±0.62 | 0.556 |
| ALT (U/L) | 53.63±6.26 | 54.05±6.52 | 0.964 | 80.93±11.54 | 113.45±17.07 | 0.146 |
| AST (U/L) | 66.3±4.77 | 79.23±8.6 | 0.218 | 89.77±7.60 | 129.55±19.65 | 0.088 |
| BUN (mmol/L) | 1.66±0.25 | 1.62±0.31 | 0.926 | 1.36±0.17 | 1.18±0.18 | 0.488 |
| HDL-C(mmol/L) | 0.95±0.14 | 1.07±0.06 | 0.461 | 1.02±0.08 | 1.17±0.10 | 0.258 |
| LDL-C (mmol/L) | 1.45±0.08 | 1.41±0.08 | 0.736 | 1.66±0.10 | 1.63±0.07 | 0.839 |
| TCHO (mmol/L) | 2.53±0.18 | 2.66±0.1 | 0.546 | 2.77±0.13 | 2.82±0.15 | 0.789 |
| TG (mmol/L) | 0.44±0.04 | 0.54±0.06 | 0.211 | 0.59±0.05 | 0.59±0.05 | 1.000 |
| TP (g/L) | 36.57±1.83 | 34.95±1.37 | 0.496 | 37.93±1.72 | 40.28±1.67 | 0.350 |

Note: GLU, glucose; ALB, albumin; ALT, alanine aminotransferase; AST, aspartate aminotransferase; BUN, blood urea nitrogen; HDL-C, high-density lipoprotein cholesterol; LDL-C, low-density lipoprotein cholesterol; TCHO, total cholesterol; TG, triglyceride; TP, total protein.

Table S4. Effects of phage cocktail on the blood routine indices of piglets

| Indexes | Day 11 | | | Day 21 | | |
|---|---|---|---|---|---|---|
| | CON | Phage | *P*-value | CON | Phage | *P*-value |
| WBC ($10^9$/L) | 18.39±3.24 | 16.07±0.81 | 0.514 | 24.05±2.94 | 22.67±0.95 | 0.672 |
| Neu ($10^9$/L) | 5.57±1.20 | 5.80±1.14 | 0.893 | 4.89±0.86 | 5.72±0.72 | 0.477 |
| Lym ($10^9$/L) | 11.93±3.41 | 9.32±1.22 | 0.486 | 16.73±2.26 | 15.12±0.91 | 0.523 |
| Mon ($10^9$/L) | 0.73±0.12 | 0.82±0.08 | 0.569 | 2.09±0.46 | 1.53±0.05 | 0.280 |
| Eos ($10^9$/L) | 0.11±0.01 | 0.10±0.02 | 0.538 | 0.25±0.06 | 0.17±0.04 | 0.301 |
| Bas ($10^9$/L) | 0.05±0.01 | 0.04±0.01 | 0.591 | 0.09±0.02[a] | 0.14±0.01[b] | 0.037 |
| Neu (%) | 33.63±5.89 | 36.08±6.63 | 0.788 | 20.52±3.38 | 25.15±2.85 | 0.319 |
| Lym (%) | 60.88±6.69 | 57.97±6.88 | 0.768 | 70.03±3.98 | 66.73±2.97 | 0.522 |
| Mon (%) | 4.53±0.84 | 5.08±0.49 | 0.583 | 8.10±1.49 | 6.80±0.30 | 0.412 |
| Eos (%) | 0.70±0.14 | 0.62±0.16 | 0.700 | 0.97±0.25 | 0.73±0.15 | 0.438 |
| Bas (%) | 0.25±0.04 | 0.25±0.04 | 1.000 | 0.33±0.05[a] | 0.58±0.08[b] | 0.027 |
| RBC ($10^{12}$/L) | 6.15±0.26 | 5.90±0.44 | 0.635 | 5.98±0.26 | 6.07±0.28 | 0.821 |
| HGB (G/L) | 114.67±6.08 | 104.17±3.33 | 0.161 | 105.67±1.74 | 104.17±1.11 | 0.485 |
| HCT (%) | 33.8±1.81 | 30.47±0.92 | 0.132 | 32.88±0.68 | 31.43±0.37 | 0.091 |
| MCV (fL) | 54.93±1.95 | 52.33±2.08 | 0.382 | 55.28±1.52 | 52.28±1.78 | 0.229 |
| MCH (pg) | 18.65±0.79 | 17.90±0.74 | 0.504 | 17.83±0.71 | 17.32±0.64 | 0.603 |
| MCHC (g/L) | 339.5±3.37 | 341.83±1.82 | 0.560 | 322.17±4.98 | 331.17±2.07 | 0.126 |
| RDW-CV (%) | 21.75±0.89 | 22.27±0.92 | 0.695 | 20.83±0.53 | 21.72±1.02 | 0.462 |
| RDW-SD (fL) | 42.62±2.13 | 41.58±0.83 | 0.666 | 40.75±1.33 | 39.83±0.99 | 0.593 |
| PLT ($10^9$/L) | 341.83±44.23 | 444.33±64.81 | 0.221 | 468.33±73.00 | 483.83±29.24 | 0.848 |
| MPV (fL) | 9.27±0.37 | 7.63±1.42 | 0.292 | 8.67±0.19 | 8.50±0.23 | 0.592 |
| PDW (%) | 15.35±0.19 | 15.15±0.12 | 0.402 | 15.65±0.11 | 15.4±0.16 | 0.228 |
| PCT (%) | 0.32±0.04 | 0.41±0.06 | 0.227 | 0.40±0.06 | 0.41±0.03 | 0.915 |

Note: WBC, white blood cells; Neu, neutrophils; Lym, lymphocytes; Mon, monocytes; Eos, Eosinophils; Bas, Basophils; RBC, red blood cells; HGB, hemoglobin; HCT, hematocrit; MCV, mean corpuscular volume; MCH, mean corpuscular hemoglobin; MCHC, mean corpuscular hemoglobin concentration; RDW-CV, red blood cell distribution width variation coefficient; RDW-SD, red blood cell distribution width standard deviation; PLT, platelet count; MPV, mean platelet volume; PDW, platelet distribution width; PCT, platelet-crit.

Note: Different superscript letters in the same row indicate significant differences between groups ($P < 0.05$).

Table S5. Effects of phage cocktail on the relative weight of organs in piglets

| Organs (%) | CON | Phage | $P$-value |
|---|---|---|---|
| Heart | 0.56±0.02 | 0.59±0.02 | 0.382 |
| Liver | 2.93±0.13 | 3.01±0.15 | 0.699 |
| Spleen | 0.28±0.02 | 0.24±0.01 | 0.136 |
| Pancreas | 0.25±0.02$^{a}$ | 0.20±0.12$^{b}$ | 0.050 |
| Kidney | 0.59±0.03 | 0.57±0.01 | 0.546 |

Note: Different superscript letters in the same row indicate significant differences between groups ($P < 0.05$).

Table S6. Effects of phage cocktail on the relative weight and length of different intestinal segments of piglets

| Segment | Indexes | CON | Phage | $P$-value |
|---|---|---|---|---|
| Jejunum | Relative length (cm/kg) | 63.49±2.46 | 68.45±1.43 | 0.103 |
| | Relative total weight (%) | 4.26±0.33 | 4.16±0.22 | 0.803 |
| | Relative net weight (%) | 3.55±0.23 | 3.56±0.17 | 0.983 |
| Ileum | Relative length (cm/kg) | 1.27±0.16 | 1.72±0.15 | 0.071 |
| | Relative total weight (%) | 0.11±0.02 | 0.14±0.01 | 0.189 |
| | Relative net weight (%) | 0.09±0.01$^{a}$ | 0.14±0.01$^{b}$ | 0.024 |
| Cecum | Relative total weight (%) | 0.46±0.10 | 0.62±0.09 | 0.271 |
| | Relative net weight (%) | 0.21±0.01 | 0.22±0.01 | 0.58 |
| Colon | Relative length (cm/kg) | 10.07±0.92 | 9.95±0.83 | 0.922 |
| | Relative total weight (%) | 2.61±0.23 | 2.35±0.17 | 0.388 |

Note: Different superscript letters in the same row indicate significant differences between groups ($P < 0.05$).

Table S7. Effects of phage cocktail on the digestive enzymes' activity of jejunal digesta

| Digestive enzyme （*U*/mgprot) | CON | Phage | $P$-value |
|---|---|---|---|
| Lipase | 0.17±0.04 | 0.12±0.02 | 0.313 |
| Trypsin | 6643.52±1655.35 | 4355.24±1389.83 | 0.313 |
| Amylase | 13.68±6.69 | 10.46±2.41 | 0.660 |

Table S8. Effects of phage cocktail on the intestinal morphology of piglets

| Indexes | CON | Phage | *P*-value |
|---|---|---|---|
| Jejunum villus height (μm) | 490.73±94.31 | 443.95±32.13 | 0.649 |
| Jejunum crypt depth (μm) | 241.98±16.59 | 241.40±15.39 | 0.980 |
| villus height/crypt depth | 2.10±0.46 | 1.85±0.13 | 0.608 |

Table S9. Effects of phage cocktail on the α diversity of fecal microbiota of piglets

| Indexes | Day 11 | | | Day 21 | | |
|---|---|---|---|---|---|---|
| | CON | Phage | *P*-value | CON | Phage | *P*-value |
| Observed | 2158.83±187.43 | 1954.67±108.35 | 0.368 | 1975.17±87.22 | 1780.50±190.35 | 0.374 |
| Chao1 | 3023.83±214.83 | 2680.03±134.98 | 0.205 | 2799.16±145.53 | 2524.54±250.00 | 0.365 |
| ACE | 3056.70±226.76 | 2748.87±137.03 | 0.272 | 2842.13±132.76 | 2545.82±259.44 | 0.333 |
| Shannon | 5.32±0.12 | 5.25±0.12 | 0.680 | 5.18±0.08 | 5.09±0.17 | 0.667 |
| Simpson | 0.98±0.005 | 0.98±0.003 | 0.831 | 0.98±0.003 | 0.97±0.005 | 0.511 |
